# Effects of Etomidate on the Steroidogenesis of Rat Immature Leydig Cells

**DOI:** 10.1371/journal.pone.0139311

**Published:** 2015-11-10

**Authors:** Hua-Cheng Liu, Danyan Zhu, Chan Wang, Hongguo Guan, Senlin Li, Cong Hu, Zhichuan Chen, Yuanyuan Hu, Han Lin, Qing-Quan Lian, Ren-Shan Ge

**Affiliations:** 1 Department of Anesthiology, The Second Affiliated Hospital & Yuying Children's Hospital of Wenzhou Medical University, Wenzhou Medical University, Wenzhou, Zhejiang, 325027, People’s Republic of China; 2 School of Pharmacy, Wenzhou Medical University, Wenzhou Medical University, Wenzhou, Zhejiang, 325035, People’s Republic of China; Imperial College London, Chelsea & Westminster Hospital, UNITED KINGDOM

## Abstract

**Background:**

Etomidate is a rapid hypnotic intravenous anesthetic agent. The major side effect of etomidate is the reduced plasma concentration of corticosteroids, leading to the abnormal reaction of adrenals. Cortisol and testosterone biosynthesis has similar biosynthetic pathway, and shares several common steroidogenic enzymes, such as P450 side chain cleavage enzyme (CYP11A1) and 3β-hydroxysteroid dehydrogenase 1 (HSD3B1). The effect of etomidate on Leydig cell steroidogenesis during the cell maturation process is not well established.

**Methodology:**

Immature Leydig cells isolated from 35 day-old rats were cultured with 30 μM etomidate for 3 hours in combination with LH, 8Br-cAMP, 25R-OH-cholesterol, pregnenolone, progesterone, androstenedione, testosterone and dihydrotestosterone, respectively. The concentrations of 5α-androstanediol and testosterone in the media were measured by radioimmunoassay. Leydig cells were cultured with various concentrations of etomidate (0.3–30 μM) for 3 hours, and total RNAs were extracted. Q-PCR was used to measure the mRNA levels of following genes: *Lhcgr*, *Scarb1*, *Star*, *Cyp11a1*, *Hsd3b1*, *Cyp17a1*, *Hsd17b3*, *Srd5a1*, and *Akr1c14*. The testis mitochondria and microsomes from 35-day-old rat testes were prepared and used to detect the direct action of etomidate on CYP11A1 and HSD3B1 activity.

**Results and Conclusions:**

In intact Leydig cells, 30 μM etomidate significantly inhibited androgen synthesis. Further studies showed that etomidate also inhibited the LH- stimulated androgen production. On purified testicular mitochondria and ER fractions, etomidate competitively inhibited both CYP11A1 and HSD3B1 activities, with the half maximal inhibitory concentration (IC_50_) values of 12.62 and 2.75 μM, respectively. In addition, etomidate inhibited steroidogenesis-related gene expression. At about 0.3 μM, etomidate significantly inhibited the expression of *Akr1C14*. At the higher concentration (30 μM), it also reduced the expression levels of *Cyp11a1*, *Hsd17b3* and *Srd5a1*. In conclusion, etomidate directly inhibits the activities of CYP11A1 and HSD3B1, and the expression levels of *Cyp11a1* and *Hsd17b3*, leading to the lower production of androgen by Leydig cells.

## Introduction

Etomidate is a carboxylated imidazole first synthesized in 1964 and reported in 1965 by Janssen Pharmaceuticals [1]. Initially developed as the anti-fungal agent, its potent hypnotic activity was found during animal testing. Etomidate has a chiral carbon, thus leading to R(+)-etomidate and S(-)-etomidate enantiomers. Studies found that the R(+)-etomidate had 10–12 fold greater hypnotic potency than S(-)-etomidate [2, 3]. Etomidate is a short acting intravenous anaesthetic agent for general anaesthesia and has an elimination half-life of 3–5  hours [4]. Its anaesthetic effects in the central nerve system are thought to be mediated by type A γ-aminobutyric acid receptors [5]. Because of its high tolerance by the cardiovascular and respiratory systems, etomidate can be used to induce anesthesia in elderly, shock or trauma patients [6].

It has several common side effects, especially for the suppression of endogenous glucocorticoid biosynthesis in adrenal glands due to the inhibition of the important mitochondrial steroidogenic enzyme cytochrome P450 11β-hydroxylase (CYP11B1), which catalyzes the production of cortisol from deoxycortisol. The enzyme is 95% homologous to the aldolase enzyme for mineralocorticoid aldosterone synthesis [7, 8]. Exposure to etomidate can result in lower serum cortisol levels up to 12  hours [9]. Previous study showed that etomidate is far more potent in inhibiting adrenal steroidogenesis than in inducing sedation and hypnosis [10]. In patients the plasma concentrations of etomidate required for hypnosis are above 200 ng/ml (1 μM), while the concentrations that begin to affect adrenal cortical production happened at much lower concentration (10 ng/ml) [11]. In cultured adrenal cells the half maximal inhibitory concentration (IC_50_) for cortisol biosynthesis is 1 nM, which is equivalent to the apparent dissociation constant for etomidate binding to membranes of these cells [12].

There are several common steps for steroid biosynthesis between adrenals and gonads, including initial steps catalyzed by mitochondrial P450 cholesterol side chain cleavage enzyme (CYP11A1) and smooth endoplasmic reticulum enzyme 3β-hydroxysteroid dehydrogenase (HSD3B). Due to these common enzymes and the common substrate, cholesterol, to begin with in both systems, it is highly likely that etomidate may also affect reproductive system. Indeed, after administration with etomidate at 0.25 mg/kg to women, it caused a significant decrease of estradiol, progesterone, 17-hydroxyprogesterone, and testosterone levels within 10 min [13]. In the male, however, though there was evidence to suggest that etomidate may affect Leydig cell testosterone production when it was added to an unpurified mice testicular cell mixture [14], the effect of etomidate on Leydig cell steroidogenesis, especially during the cell maturation process, is not well established.

**Fig 1:**
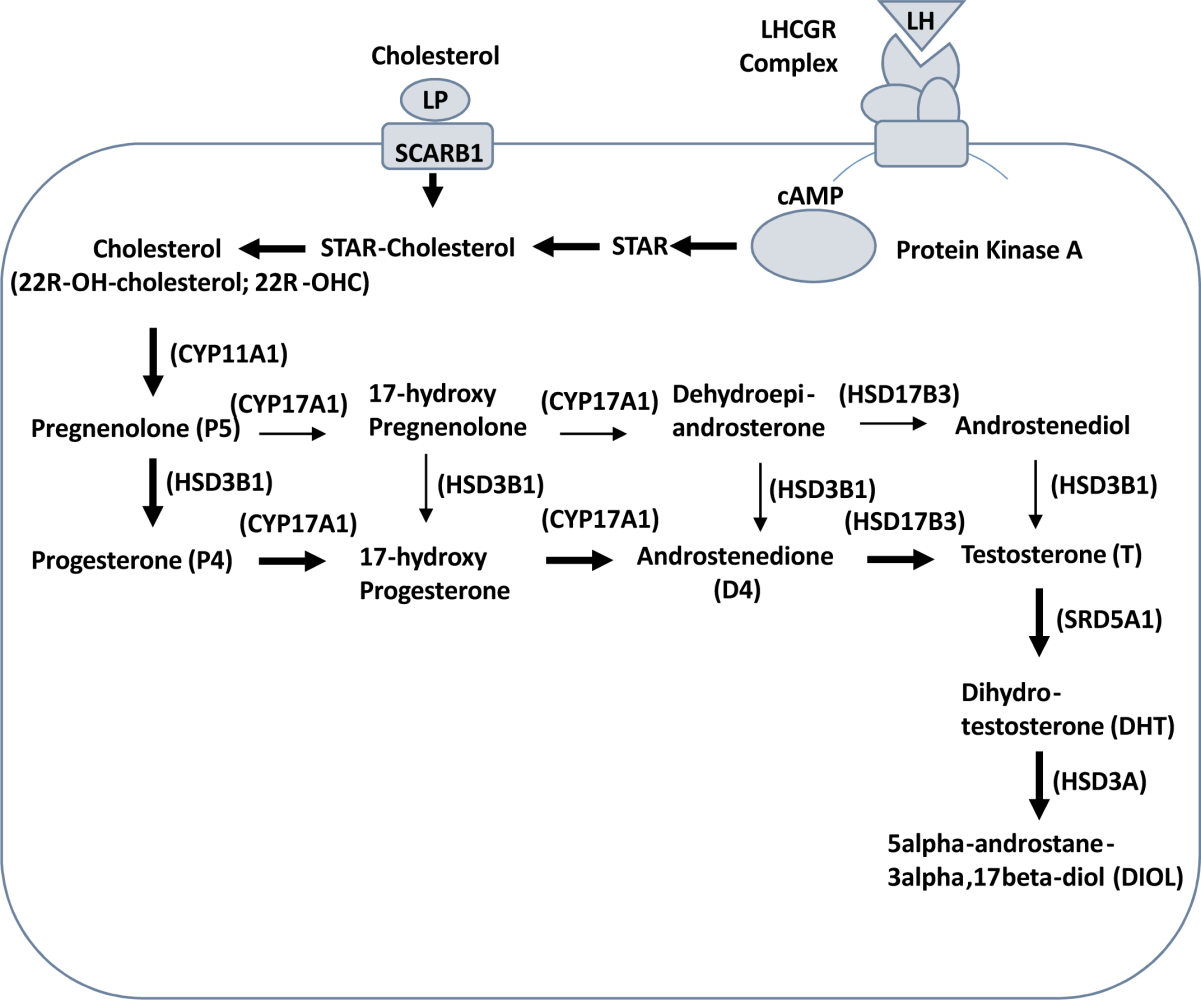
Steroidogenic pathway in rat immature Leydig cells. Cholesterol is transported into Leydig cells via lipoprotein (LP)-receptor (SCARB1). When LH binds to luteinizing hormone receptor (LHCGR) to induce LHCGR-complex interaction leading to cAMP cascade, the latter activates protein kinase A, which induces the expression of steroidogenic acute regulatory protein (STAR). STAR is the rate-limiting step to transport intracellular cholesterol into mitochondrial inner membrane. P450 cholesterol side chain cleavage enzyme (CYP11A1); 3β-hydroxysteroid dehydrogenase 1 (HSD3B1); 17α-hydroxylase/C17,20-lyase (CYP17A1); 17β-hydroxysteroid dehydrogenase 3 (HSD17B3); 5α-reductase 1 (SRD5A1); 3α-hydroxysteroid dehydrogenase (HSD3A).

The present study will address the effects of etomidate on steroidogenesis in purified male rat immature Leydig cells. The testosterone homeostasis relies on the balance of testosterone biosynthesis and metabolism. Rat immature Leydig cells were good model for the study of testosterone biosynthesis and metabolism, because these cells contain testosterone biosynthetic enzymes: CYP11A1 (encoded by *Cyp11a1*), HSD3B1 (encoded by *Hsd3b1*), 17α-hydroxylase/17,20-lyase (CYP17A1, encoded by *Cyp17a1*), and 17β-hydroxysteroid dehydrogenase 3 (HSD17B3, encoded by *Hsd17b3*) as well as testosterone metabolizing enzymes 5α-reductase 1 (SRD5A1, encoded by *Srd5a1*) and 3α-hydroxysteroid dehydrogenase (HSD3A, encoded by *Akr1c14*). Cholesterol is the initial substrate used by CYP11A1 and is converted into pregnenolone in the mitochondria [15]. Pregnenolone is diffused out of mitochondria and is used by microsomal enzyme HSD3B1 to convert into progesterone, and the latter is being converted into androstenedione and testosterone sequentially by CYP17A1 and HSD17B3 [16, 17]. The testosterone metabolizing enzyme SRD5A1 in the immature Leydig cells converts testosterone into more potent androgen dihydrotestosterone (DHT). DHT is further catalyzed by HSD3A into 5α-androstane-3α,17β-diol (DIOL) [17], which is a weak androgen (See Fig 1). In rat immature Leydig cells, the DIOL is the major androgen and accounts for 80% of total androgens produced and testosterone accounts for 10% [17]. The steroid substrate cholesterol can be transported into Leydig cells via lipoprotein (LP)-receptor (SCARB1, encoded *Scarb1*). Rat immature Leydig cells are responsive to the luteinizing hormone (LH) secreted by pituitary. When LH binds to luteinizing hormone receptor (LHCGR, encoded by *Lhcgr*) to induce LHCGR-complex interaction leading to cAMP cascade, the latter activates protein kinase A, which induces the expression of steroidogenic acute regulatory protein (STAR, encoded by *Star*). STAR is the rate-limiting step to transport intracellular cholesterol into mitochondrial inner membrane.

## Materials and Methods

### Chemicals and animals

[^3^H]Pregnenolone, [^3^H]progesterone, [^3^H]androstenedione, [^3^H]testosterone, [^3^H]dihydrotestosterone were purchased from DuPont-New England Nuclear (Boston, MA). Unlabeled pregnenolone, progesterone, 17α-hydroxyprogesterone, androstenedione and testosterone were obtained from Steraloids (Newport, RI). Etomidate was purchased from Sigma (St. Louis, MO). Male Sprague-Dawley rats (30-day-old) were purchased from Shanghai Animal Center (Shanghai, China). All animal procedures were approved by the Institutional Animal Care and Use Committee of Wenzhou Medical University and were performed in accordance with the Guide for the Care and Use of Laboratory Animals.

### Immature Leydig cell isolation

Eighteen 35-day-old male Sprague Dawley rats were sacrificed by asphyxiation with CO_2_. Testes were removed and Leydig cells were purified as described previously [17]. In brief, animals were sacrificed in CO_2_ tank, testes were removed, perfused with collagenase (0.1 mg/ml) via testicular artery, digested with collagenase (0.25 mg/ml) and DNase (0.25 mg/ml) for 15 min, filtered with nylon mesh, and the cells were separated under Percoll gradient. The cells with density of 1.070–1.088 g/ml were collected and washed. Purities of Leydig cell fractions were evaluated by histochemical staining for HSD3B activity, with 0.4 mM etiocholanolone as the steroid substrate [18]. The purities of Leydig cells were around 95% consistently.

### Leydig cell culture

After isolation, the purified immature Leydig cells were seeded into 24-well culture plated with cell density of 0.05 × 10^6^ cells/well. Leydig cells were cultured in 0.5 ml DMEM:F12 medium (phenol-free) without (basal) or with hormone and signalling substances, 10 ng/ml LH and 10 mM 8-bromo-cAMP (8Br-cAMP), 20 μM of various steroid substrates including 25R-OH-cholesterol (25R-OHC), pregnenolone, progesterone, androstenedione, testosterone, dihydrotestosterone for 3 hours in the presence of 30 μM etomidate (etomidate was dissolved in ethanol and ethanol was the control). Because 8Br-cAMP can penetrate the cell membrane, therefore it is used to replace the intracellular cAMP, which is impermeable. 25R-OHC, pregnenolone, progesterone, androstenedione, testosterone, and dihydrotestosterone were used as the respective substrate of the enzyme: CYP11A1, HSD3B1, CYP17A1, HSD17B3, SRD5A1, and HSD3A. Because 25R-OHC can readily penetrate cell and mitochondrial membrane, it is used to replace cholesterol as substrate for CYP11A1. Media were collected for DIOL and testosterone assay after incubation.

### Preparation of mitochondrial, cytosol and microsomal proteins

Mitochondrial, cytosol and microsomal preparations of rat testes were done as described previously [19]. Testes (from 35-day-old Sprague Dawley male rats) were homogenized in cold 0.01mM phosphate buffered saline (PBS) containing 0.25 mM sucrose and centrifuged at 700 × g for 30 min. The supernatants were transferred to new tubes and centrifuged at 10,000 × g for another 30 min and washed twice to collect mitochondrial pellet. Supernatants then were further centrifuged at 105,000 × g for 1 h twice to collect microsomal pellet and supernatant as cytosol. Pellets were resuspended and protein concentrations in these fractions were measured using the Bio-Rad Protein Assay Kit (cat# 500–0006; Bio-Rad, Hercules, CA) according to manufacturer’s protocol. Mitochondria were used for CYP11A1 measurement. Microsomes were used for measurement of HSD3B1, CYP11A1, HSD17B3, and SRD5A1 enzyme activities. Cytosol was used for HSD3A measurement. According to pilot studies, the enzymatic reactions below were all still linear by the end of the assays with the substrates used less than 10%. The component of the final assay mixture is listed in Table 1. The final mixture volume is 250 μl. We used 34°C for enzyme activity assay is due to that fact that in mammals (human and rat) the *in vivo* testicular (Leydig cells) temperature is 34°C.

**Table 1:** Enzymatic assays of HSD3B1, CYP17A1, HSD17B3, SRD5A1 and HSD3A.

Enzyme	Substrates (μM)	Co-factors (mM)	Microsome /Cytosol (μg)	Time (min)
HSD3B1	P5 (0.2)	NAD^+^ (0.2)	microsome (10)	90
CYP17A1	P4 (1)	NADPH (0.2)	microsome (20)	60
HSD17B3	D4 (0.2)	NADPH (0.2)	microsome (90)	60
SRD5A1	T (2)	NADPH (0.2)	microsome (90)	60
HSD3A	DHT (2)	NADPH (0.2)	cytosol (90)	60

### CYP11A1 assay

CYP11A1 activity in testicular mitochondria was assayed using 25R-OHC as a substrate and pregnenolone as a product. Briefly, 25R-OHC (2 μM) was dissolved in ethanol, with final ethanol concentration in the reaction solution no more than 0.2%. The substrate concentration was selected based on the Km value in a pilot study. In order to determine half maximal inhibitory concentration (IC_50_), 60 min reactions were initiated by addition of rat (10 μg) testis mitochondrial protein in the presence of varying concentrations of etomidate (0.01–100 μM). In separated assays to determine the inhibitory mechanism of etomidate, concentrations of 25R-OHC ranging from 0.002 to 10 μM were added to reaction mixture containing 10 μg rat testis mitochondria and 10–50 μM etomidate, and the mixtures were incubated at 34^°^C for 60 min. By end of incubation, the product, pregnenolone, was assayed by RIA kit. The percentage conversion of 25R-OHC to pregnenolone was calculated by pregnenolone from the substrate.

### Enzymatic assays of HSD3B1, CYP17A1, HSD17B3, SRD5A1 and HSD3A

The testicular microsomal and cytosol enzymatic assays were done as described previously [20]. The detailed conditions for each enzymatic assay were listed in the Table 1. Briefly, the mixture (250 μl) of the substrates (0.2–2 μM), the tracers (40,000 dpm) and co-factors (NAD^+^ or NADPH, up to 0.2 mM) were incubated with certain amounts of enzymes (microsomal or cytosol fractions) for 60–90 min at 34 C (the temperature of normal testis). For some reactions, etomidate was added as inhibitor (up to 100 μM). By the end of incubations, the reactions were stopped by adding 2 ml ice cold ether. The steroids were extracted, and the organic layer was dried under nitrogen. Steroids were separated chromatographically on thin layer plates (Baker-Flex Silica Gel IB-F coated with 200 μm analytical layer and fluorescent indicator, 20 x 20 cm, Thomas Scientific, Swedesboro, NJ) in chloroform and methanol (97:3, v/v), and radioactivity was measured using a scanning radiometer (System AR2000, Bioscan Inc., Washington, DC)(S1 Fig). The percentage conversion of the substrates to products was calculated by dividing the radioactive counts identified as products by the total counts of substrates plus products.

### Assay of P5, DIOL and T concentrations

Pregnenolone, DIOL and testosterone concentrations in the medium was measured with a tritium-based radioimmunoassay (RIA) as described [17], using the commercial RIA kits (IBL, USA). Inter-assay variation of the pregnenolone, DIOL and testosterone was within 15%.

### Extraction of RNA and teal-time PCR (qPCR)

Total RNAs were extracted from immature Leydig cells using Trizol reagent (Invitrogen, Carlsbad, CA, USA) according to the manufacturer's instruction. The ten Leydig cell genes and their primers were used as described previously [21, 22]. These genes are membrane receptor genes including *Lhcgr*, cholesterol transporting genes including *Scarb1* and *Star*, and steroidogenic enzyme genes including CYP11A1 (*Cyp11a1*), HSD3B1 (*Hsd3b1*), CYP17A1 (*Cyp17a1*), HSD17B3 (*Hsd17b3*), SRD5A1 (*Srd5a1*), and HSD3A (*Akr1c14*). HSD3A, or more commonly, 3α-HSD, is coded by *AKR1C4* gene in human. The analogue of *AKR1C4* in the rat is *Akr1c14*. The relative mRNA levels of targeted genes were normalized to *Rps16* (internal control gene). The RNA was reversely transcribed into cDNA using random hexamers and MMLV reverse transcriptase by the kit (Promega, CA) according to the manufacturer’s instruction. Q-PCR was carried out in a 25-μl reaction volume with SYBR Green detection system (Bio-Rad Laboratories, Inc., Hercules, CA, USA). Reactions were run on a Bio-Rad qPCR system (Bio-Rad Laboratories, Inc., Hercules, CA, USA) for up to 40 cycles and the melting curves were always checked afterward.

### Statistics

Data were subjected to analysis by student t-test to identify significant differences whenever two groups (a single concentration of etomidate vs. control) were compared. Data were subjected to analysis by Kruskal-Wallis test followed by ad hoc Dunnett's multiple comparisons to identify significant differences between the tested group and the controls whenever there were three or more groups (multiple concentrations of etomidate vs. control) were compared. The IC_50_ was calculated using GraphPad (Version 5, GraphPad Software Inc., San Diego, CA) using nonlinear regression of curve fit with one-site competition. Lineweaver-Burk analysis was used to determine the mode of inhibition. All experiments were repeated 3–5 times, depending on the experiments. All data are expressed as means ± SEM. Differences were regarded as significant at *P* < 0.05.

### Results

#### Effects of etomidate on androgen production in rat immature Leydig cells

**Fig 2:**
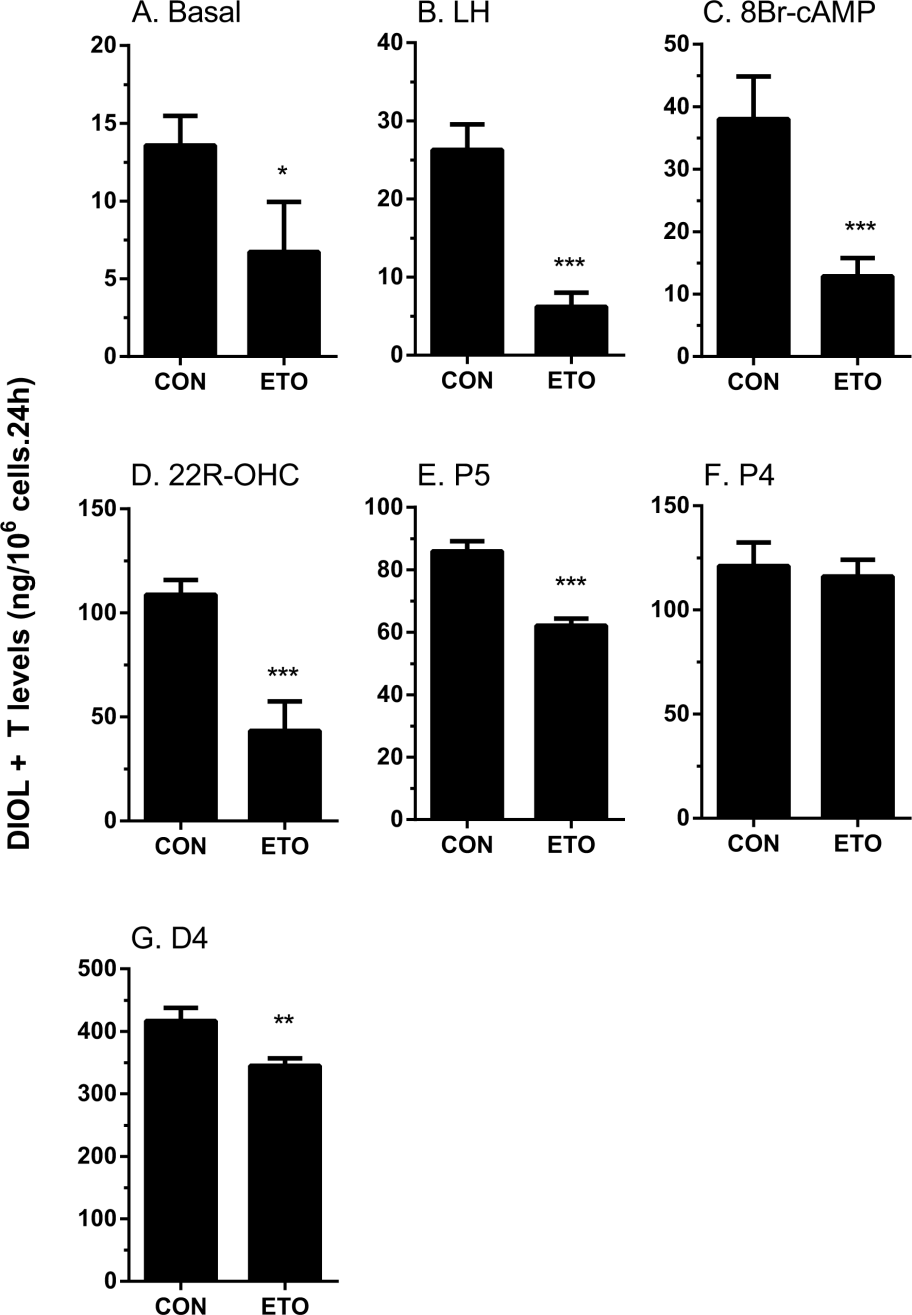
Effects of etomidate on androgen production by rat immature Leydig cells. Rat immature Leydig cells were cultured without or with luteinizing hormone (LH), 8bromo-cAMP (8Br-cAMP), 25R-OH-cholesterol (25R-OHC), pregnenolone (P5), progesterone (P4), and androstenedione (D4) in combination with 30 μM etomidate (ETO) for 3 hrs. Medium 5α-androstanediol (DIOL) and testosterone (T) levels were measured. Mean ± SEM, n = 4; *, **, *** indicate significant difference when compared to control at P < 0.05, 0.01, 0.001, respectively.

**Fig 3:**
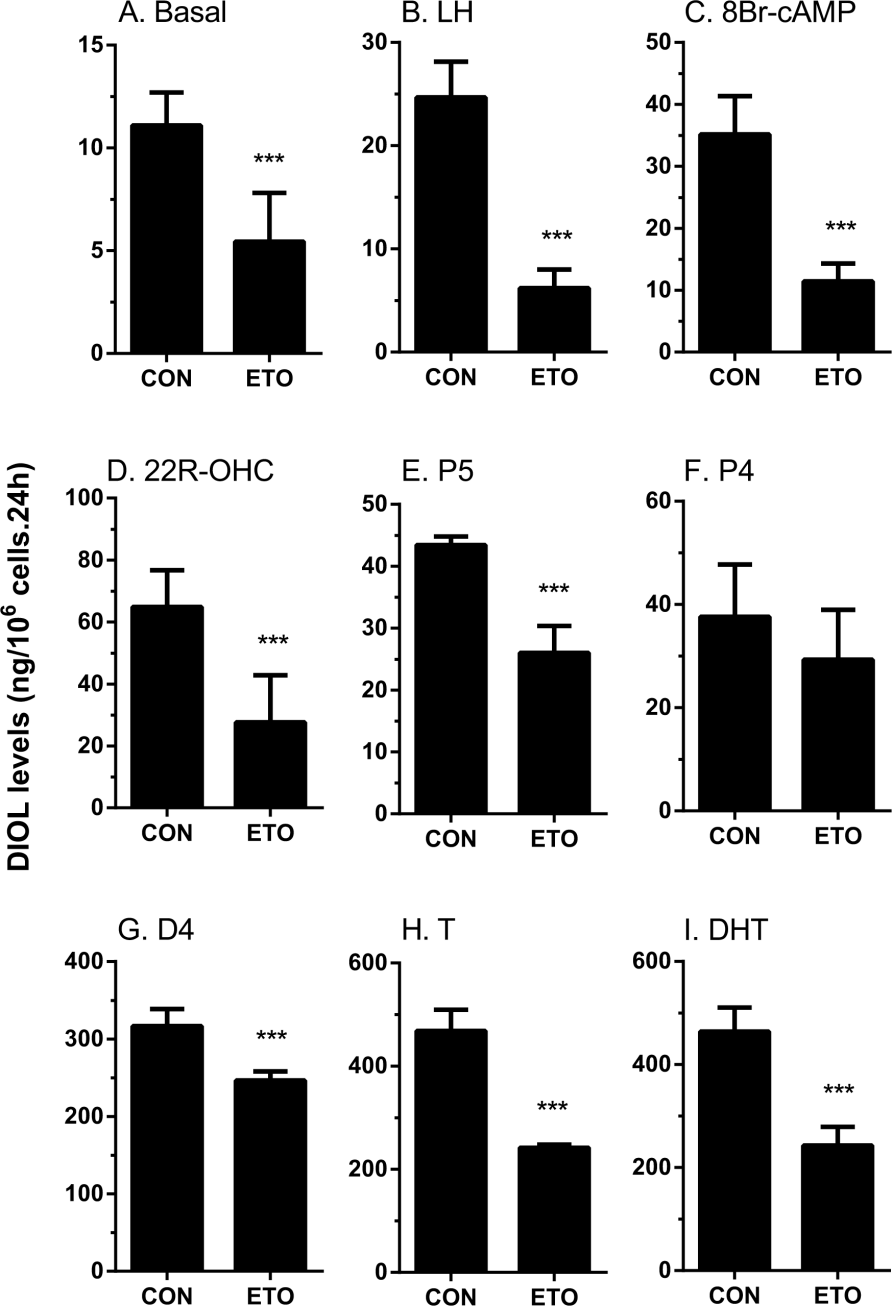
Effects of etomidate on 5α-androstanediol production by rat immature Leydig cells. Rat immature Leydig cells were cultured without or with luteinizing hormone (LH), 8bromo-cAMP (8Br-cAMP), 25R-OH-cholesterol (25R-OHC), pregnenolone (P5), progesterone (P4), and androstenedione (D4), testosterone (T) and dihydrotestosterone (DHT) in combination with 30 μM etomidate (ETO) for 3 hrs. Medium 5α-androstanediol (DIOL) levels were measured. Mean ± SEM, n = 4; *, **, *** indicate significant difference when compared to control at P < 0.05, 0.01, 0.001, respectively.

**Fig 4:**
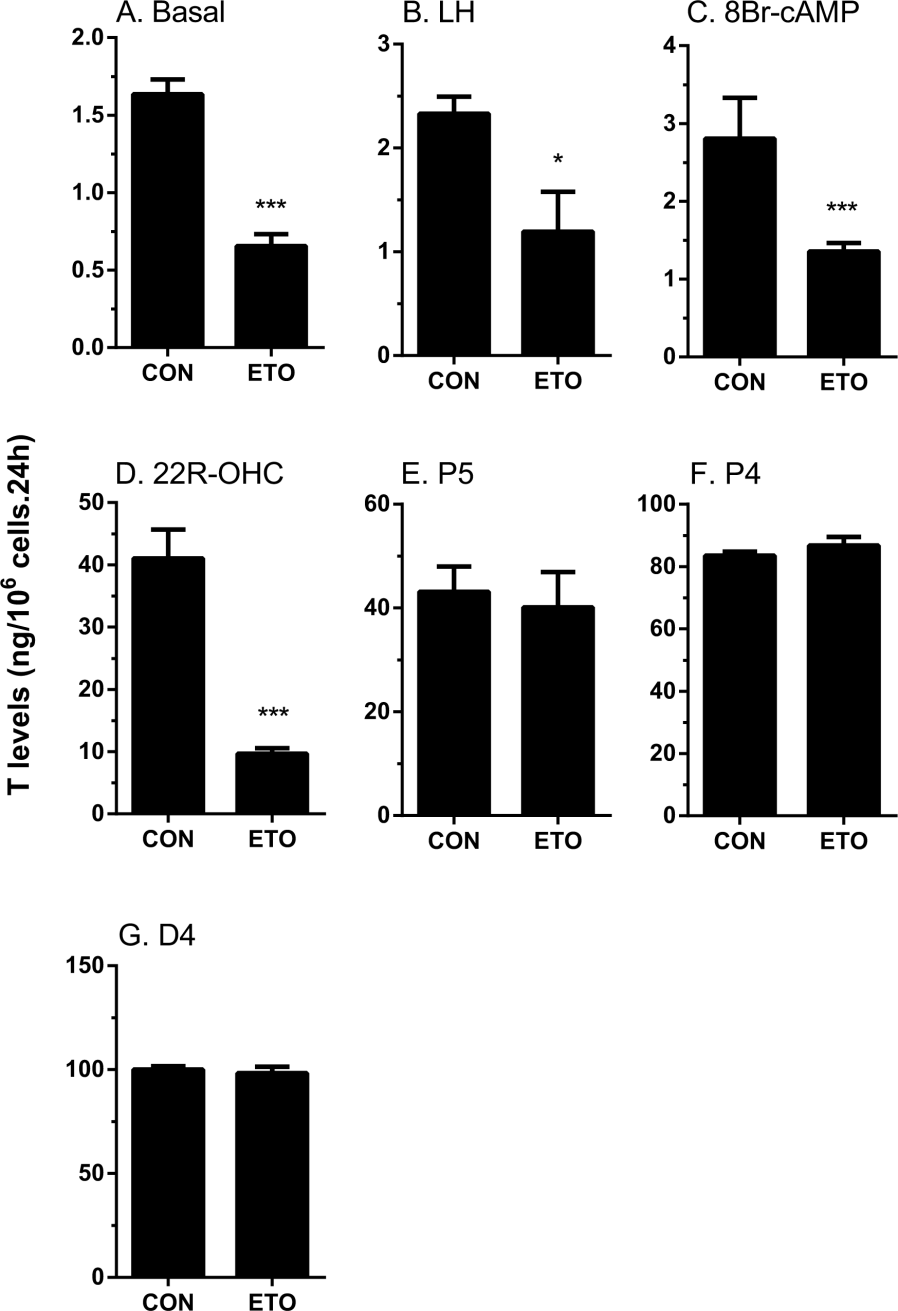
Effects of etomidate on testosterone production by rat immature Leydig cells. Rat immature Leydig cells were cultured without or with Luteinizing hormone (LH), 8bromo-cAMP (8Br-cAMP), 25R-OH-cholesterol (25R-OHC), pregnenolone (P5), progesterone (P4), and androstenedione (D4) in combination with 30 μM etomidate (ETO) for 3 hrs. Medium testosterone (T) levels were measured. Mean ± SEM, n = 4; *, **, *** indicate significant difference when compared to control at P < 0.05, 0.01, 0.001, respectively.

The rat immature Leydig cell primarily produces DIOL, because it contains androgen metabolizing enzymes (SRD5A1 and HSD3A) [17] (Fig 1). To test the effects of etomidate on androgen biosynthesis and metabolism, we added hormone (LH, 10 ng/ml), signaling compound (8Br-cAMP, 10 mM) and steroidogenic enzyme substrates, including those of CYP11A1 (25R-OHC, 20 μM), HSD3B1 (pregnenolone, P5, 20 μM), CYP17A1 (progesterone, P4, 20 μM), HSD17B3 (androstenedione, D4, 20 μM), SRD5A1 (testosterone, T, 20 μM), and HSD3A (DHT, 20 μM), and measured the medium DIOL and T and then compared them with the control (no treatment, basal). As shown in Fig 2 (DIOL plus T), Fig 3 (DIOL) and Fig 4 (T), and as expected, under basal, LH and 8Br-cAMP stimulations, DIOL was the major androgen, which accounted for 9 folds over T level. At basal, LH and 8Br-cAMP-stimulated conditions, etomidate consistently inhibited T/DIOL productions. Because the inhibitions were comparable between LH and 8Br-cAMP stimulations, it suggests that the inhibition site(s) may be beyond the LH signaling cascade. To further elucidate the specific cascades by which etomidate may affect androgen productions, we tested all the enzymatic cascades by providing the cells with different substrates that start the pathway reactions from different points. After addition of 25R-OHC, P5, P4, and D4 as substrates, the final androgen output (T+DIOL), was still lower with 25R-OHC, but gradually increased stepwise toward to controls when P5 and P4 were used as substrates (Fig 2F). It suggests that the major inhibition is between the cascades from cholesterol to progesterone (CYP11A1 and HSD3B). Interestingly, though there is a small reduction when D4 was used as substrate (Fig 2G), the total androgen productions did not change when P4 was used as substrate (Fig 2F). It suggests that in these late stages, the conversion rate from D4 to T/DIOL is much more efficient than the rate from P4 to D4 such that a small inhibition from D4 to T/DIOL apparently did not limit the androgen production when P4 used as substrate (Fig 2F).

When T and DIOL were considered separately, the results were also intriguing. With P5 and D4 as substrates, T productions were not affected (Fig 4E and 4G) while the production of DIOL were significantly reduced (Fig 3E and 3G). This suggests that in addition to the T synthetic cascades, the T metabolizing cascades (SRD5A1 and HSD3A) were also affected by etomidate. This is confirmed by observations that when T and DHT were used as substrates, DIOL production were significantly reduced (Fig 3H and 3I).

#### Dose-dependent inhibitions of etomidate on androgen production in rat Leydig cells under basal condition

**Fig 5:**
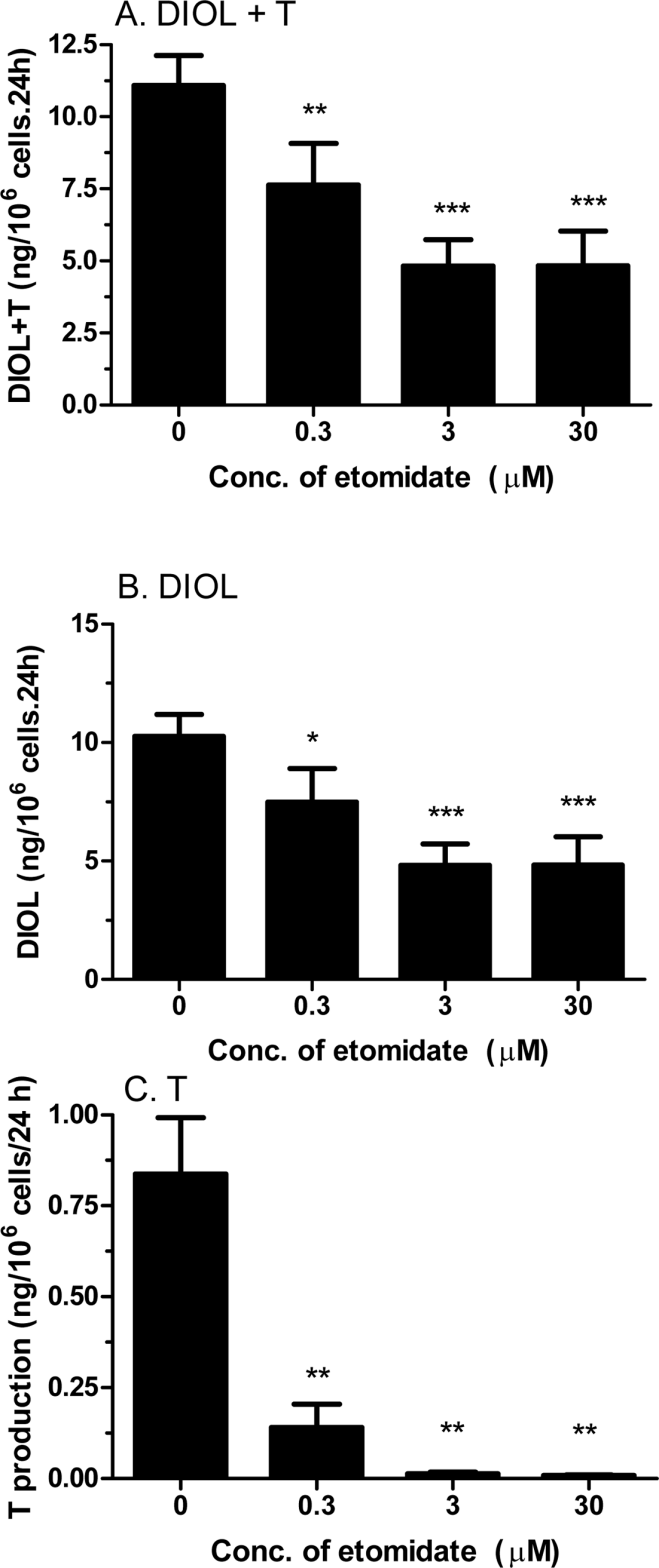
Concentration dependent effects of etomidate on basal androgen production by rat immature Leydig cells. Rat immature Leydig cells were cultured with 0.3–30 μM etomidate (ETO) for 3 hrs. Medium testosterone (T) levels were measured. Mean ± SEM, n = 4; *, **, *** indicate significant difference when compared to control at P < 0.05, 0.01, 0.001, respectively.

Since etomidate (30 μM) is capable of reducing androgen productions under unstimulated condition (Basal), we then tested how the dosages of etomidate may affect androgen productions. With the three concentrations we tested, etomidate inhibited both T and DIOL productions in a dose-dependent manner (Fig 5). The effective concentration to inhibit T production may go even lower than 0.3 μM. Interestingly, under unstimulated condition, etomidate may inhibit the T synthetic cascades much more efficient than T metabolizing cascades since the T productions have much severer reductions than DIOL at every concentrations tested (Fig 5). These results suggest that etomidate is a potent inhibitor of androgen biosynthesis, even at unstimulated condition.

#### Concentration dependent effects of etomidate on the expression levels of genes related with androgen biosynthesis

**Fig 6:**
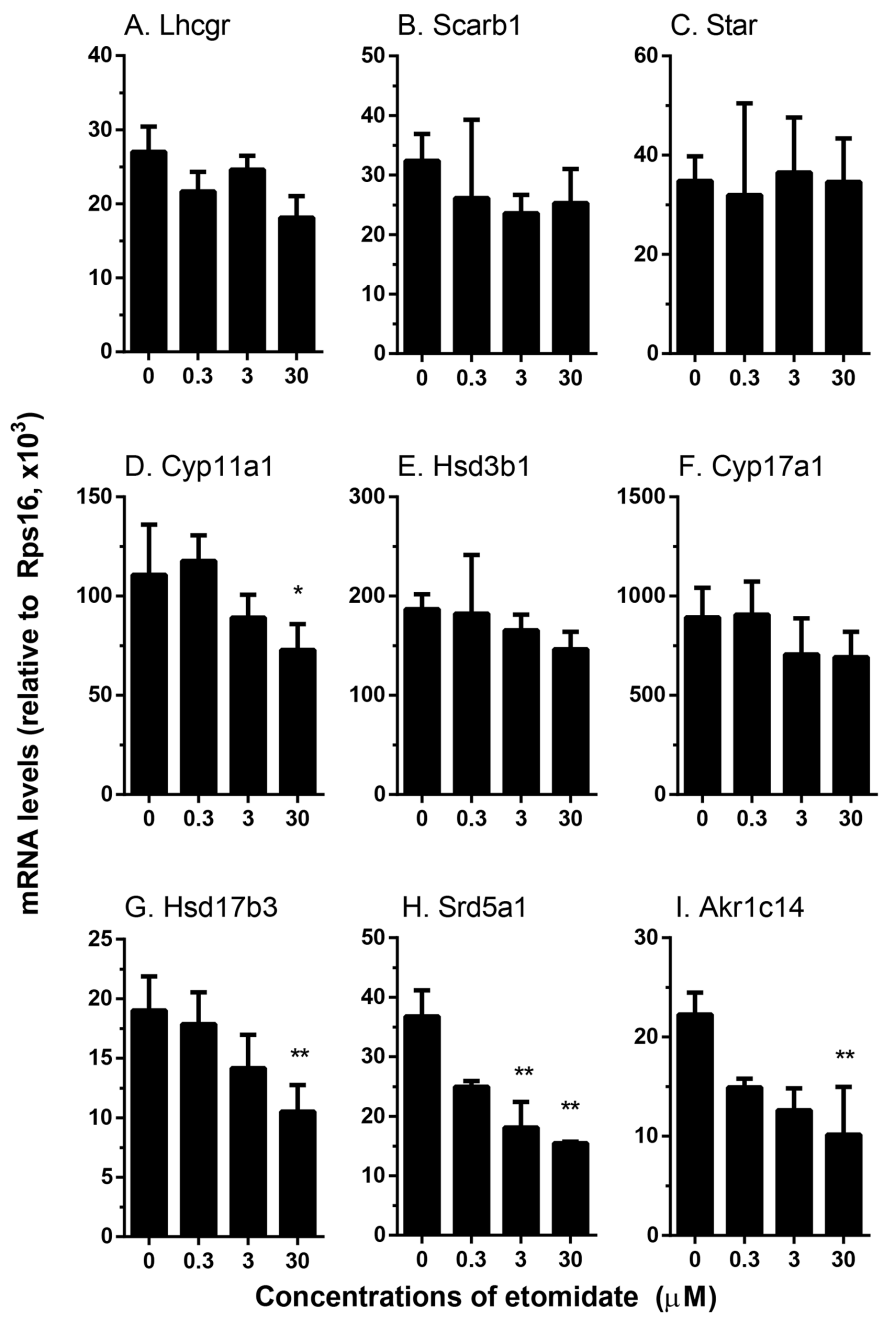
Effects of etomidate on the expression levels of steroidogenesis related genes in rat immature Leydig cells. Rat immature Leydig cells were cultured with 0.3–30 μM etomidate (ETO) for 3 hrs. The expression levels of steroidogenesis-related genes were measured and calculated relatively to *Rps16*, the internal control. Mean ± SEM, n = 4; *, **, *** indicate significant difference when compared to control at P < 0.05, 0.01, 0.001, respectively.

We examined the effects of etomidate on the expression levels of genes that are related with androgen biosynthesis and metabolism (Fig 6). Statistically, we found at ≥ 3 μM it significantly downregulated the *Srd5a1* level. At the highest concentration (30 μM) tested, etomidate also significantly downregulated *Cyp11a1*, *Hsd17b3* and *Akr1c14* levels. However, at this highest concentration, it did not affect the expression levels of *Lhcgr*, *Scarb1*, *Star*, *Hsd3b1*, and *Cyp17a1*. These results suggest that etomidate is also capable of interfering specific steroidogenic protein productions.

#### The direct inhibition on androgen biosynthetic and metabolizing enzyme activities by etomidate

**Fig 7:**
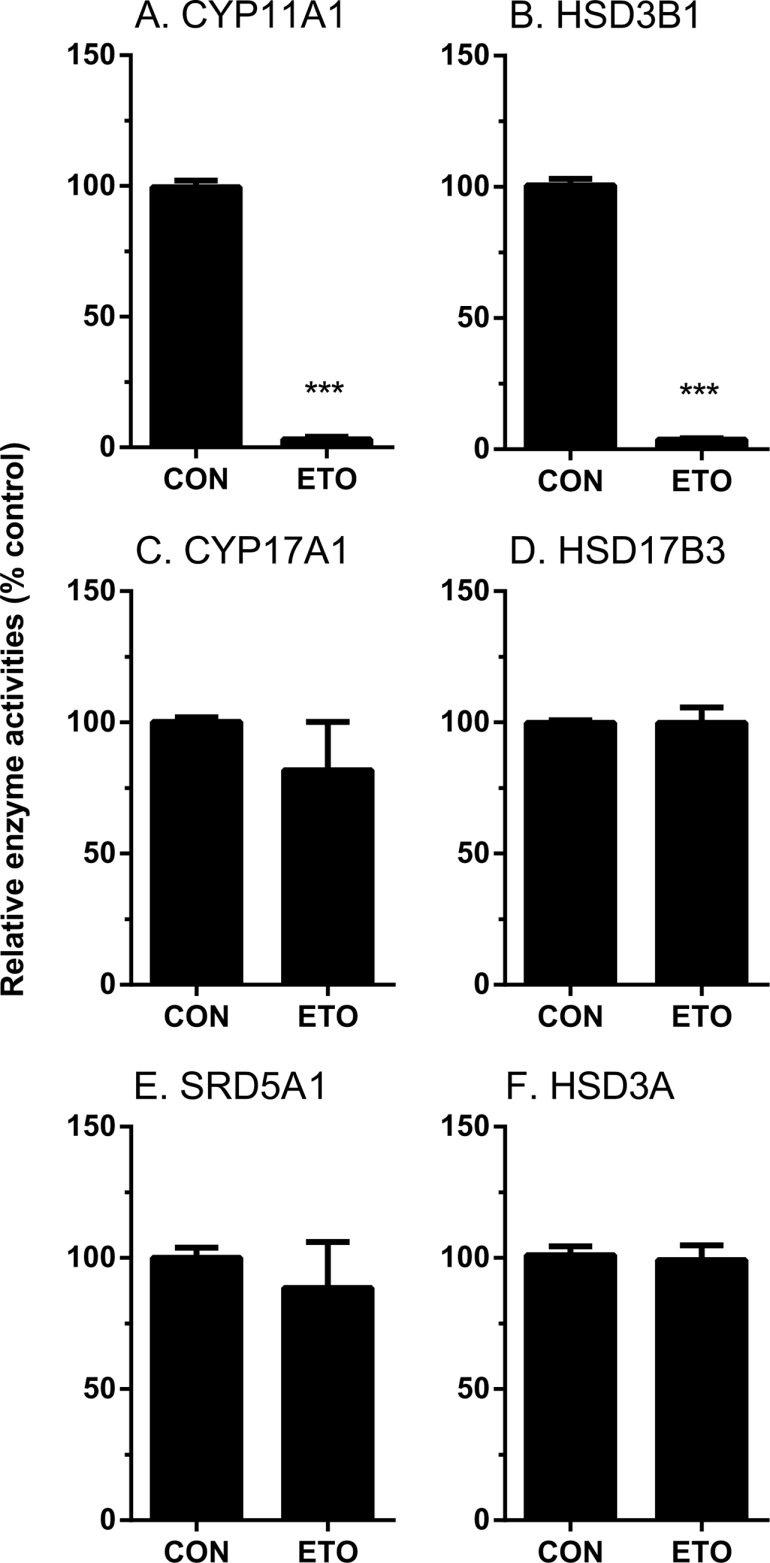
Direct effects of etomidate on androgen biosynthetic and metabolizing enzyme activities in rat testes. Rat testis enzymes were measured with 100 μM etomidate (ETO). The % activity of control was calculated. Mean ± SEM, n = 4; *, **, *** indicate significant difference when compared to control at P < 0.05, 0.01, 0.001, respectively.

We tested whether etomidate also directly inhibited androgen biosynthetic (CYP11A1, HSD3B1, CYP17A1 and CYP17A1) and metabolizing enzyme (SRD5A1 and HSD3A) activities. As shown in Fig 7, at 100 μM etomidate significantly inhibited CYP11A1 and HSD3B1 activities, while it had no effects on other enzyme activities. We further explored the concentration dependent inhibition of CYP11A1 and HSD3B1 activities, and found that the IC_50_ values for CYP11A1 and HSD3B1 were 12.62 ± 0.117 μM and 2.75 ± 0.085 μM, respectively (Table 2, Fig 7).

**Table 2:** The direct effects of etomidate on steroidogenic enzymes.

Enzyme names	IC_50_ values (μM)	% inhibition at 30 μM
CYP11A1	12.62 ± 0.117	95%
HSD3B1	2.75 ± 0.085	95%
CYP17A1	ND	NI
HSD17B3	ND	NI
SRD5A1	ND	NI
HSD3A	ND	NI

Mean ± SEM, n = 4; ND = not detected; NI = no inhibition.

#### The inhibitory modes for CYP11A1 and HSD3B by etomidate

**Fig 8:**
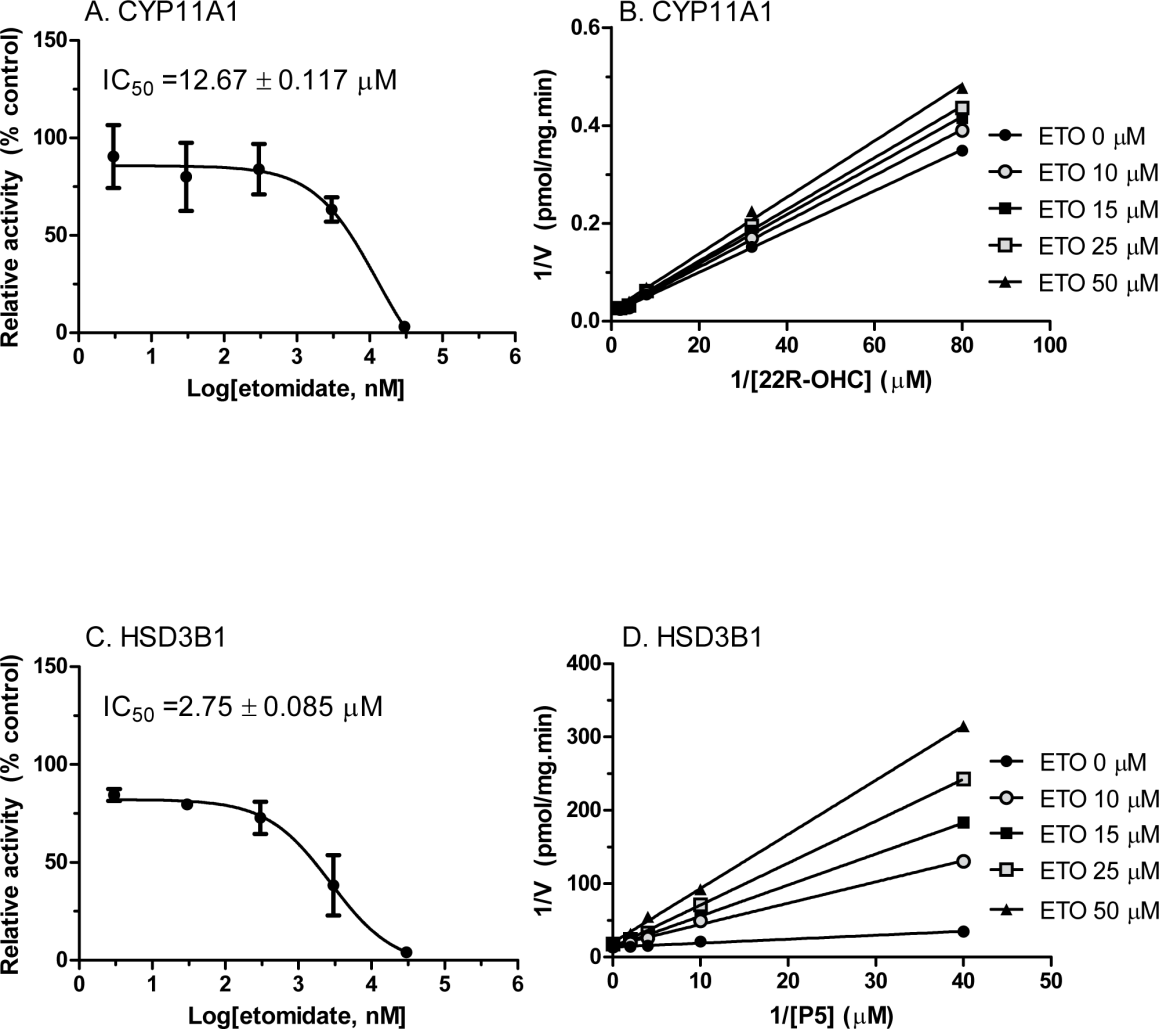
The IC_50_ values of etomidate on CYP11A1 and HSD3B1 enzymes as well as the inhibitory mode. Rat testis CYP11A1 and HSD3B1 activities were measured with various concentration of etomidate (ETO). The % activity of control was calculated. Lineweaver–Burk plot for rat CYP11A1 and HSD3B1 in the presence of steroid substrates. Panel A and C show the IC_50_ values of etomidate on CYP11A1 or HSD3B1, respectively. Panel B and D show 1/V versus 1/[25R-OHC] or 1/[P5], respectively. Mean ± SEM, n = 4.

We determined the mode of inhibition for CYP11A1 when the substrate 25R-OHC was used, and found that etomidate competitively inhibited CYP11A1 activity (Fig 8B), indicating that etomidate binds to steroid active site. Then we tested the mode of inhibition for HSD3B1 when the substrate pregnenolone (P5) was used, and found that etomidate competitively inhibited HSD3B1 activity (Fig 8D), indicating that etomidate binds to steroid active site of HSD3B1. When the cofactor NAD^+^ was used as substrate, it uncompetitively inhibited the enzyme activity, suggesting that etomidate may only bind to the complex of enzyme-NAD^+^ complex, instead of enzyme itself (S2 Fig).

## Discussion

The testosterone homeostasis in rat immature Leydig cells primary depends on the four testosterone biosynthetic enzymes (CYP11A1, HSD3B, CYP17A1, and HSD17B3) and two testosterone metabolizing enzymes (SRD5A1 and HSD3A). In the present study, we found that etomidate can, at one hand, dose-dependently inhibit the activities of two key testosterone synthetic enzymes CYP11A1 and HSD3B1. At another hand, etomidate is also capable of dose-dependently down-regulating the expression levels of the two enzymes (*Cyp11a1* and *Hsd17b3)*. Inhibitions in these key synthetic enzymes surely could contribute to the inhibition of testosterone production by etomidate. However, etomidate was also capable of inhibiting the expression levels of testosterone-metabolizing enzymes (*Srd5a1* and *Akr1c14*). Apparently, the inhibitions in synthetic pathway may play more dominantly role than in the metabolic process because even in the face of reductions in metabolic process, testosterone was still reduced. Overall, these results suggest that etomidate may impact multiple cascades in the steroidogenic pathway, which may contribute to the reductions in both T and the final product DIOL.

Etomidate directly inhibited CYP11A1 enzyme activity located in mitochondrial inner membrane with IC_50_ value of 12.67 μM (Fig 8). Therefore, it is a moderate inhibitor of this enzyme. However, etomidate did not inhibit another P450 enzyme (CYP17A1), which is located in smooth endoplasmic reticulum. Interestingly, etomidate has been reported to inhibit another mitochondrial P450 enzyme, CYP11B1, an adrenal enzyme. The inhibition of CYP11B1 led to the increase of the 11-dehydrocortisol and the decrease of cortisol and mineralocorticoid aldosterone [7, 9]. Many studies found that a single or continuous administration of etomidate led to the inhibition of adrenal function for 12–36 hours and this inhibition was recovered after use of this drug [7, 9]. These studies suggest that the direct blockage of the mitochondrial P450 enzymes probably play a major role in the process. Mitochondrial P450 enzymes belong to type I P450 enzyme family, which relies on the electron-transferring system of two electron transfer proteins: adrenodoxin reductase and adrenodoxin [23, 24]. CYP11A1, with these electron transfer proteins constitute the cholesterol side-chain cleavage complex. However, smooth endoplasmic reticulum P450 enzyme such as CYP17A1 belongs to the type II P450 enzyme family, which relies on the P450 reductase for the electronic transferring [15]. In the testing tube, the reconstituted CYP11A1 was also found sensitive to etomidate inhibition. [12], though it is less sensitive to etomidate when compared to CYP11B1. It is still unknown whether any of these differences: locations, electron transfer proteins, P450 protein themselves or the combination of all, may explain the differences in the sensitivities of the two enzymes.

Etomidate also directly inhibited HSD3B1 activity with IC_50_ value of 2.75 μM (Fig 8). This inhibition showed more potent than its action of CYP11A1. This inhibitory activity of etomidate was competitive in nature when steroid substrate pregnenolone was used (Fig 8D), indicating that etomidate binds to steroid active site of this enzyme. However, HSD3B1 is an enzyme with double substrates, pregnenolone and NAD^+^. We investigated the inhibitory activity of etomidate when cofactor NAD^+^ was used as a variable. The result showed that etomidate inhibited the enzyme activity uncompetitively, indicating that etomidate may binds to the NAD^+^-HSD3B1 complex, instead of HSD3B1 itself (S2 Fig).

The treatment of etomidate also significantly inhibited the expression levels of some testosterone biosynthetic enzymes and metabolizing enzymes. The inhibitory potencies were: *Srd5a1* (encoding SRD5A1) at 3 μM > *Cyp11a1* (encoding CYP11A1), *Hsd17b3* (HSD17B3) and *Alk1c14* (encoding HSD3A) at 30 μM. However, even at the highest concentration tested (30 μM), etomidate had no effects on *Hsd3b1* and *Cyp17a1* and other important Leydig cell specific genes (*Lhcgr*, *Scarb1* and *Star*) that are related to the androgen production. At present, though our study shows that 3 hour exposure is capable of inhibiting the mRNA levels of some of the key steroidogenic enzymes, the evidence that etomidate can affect steroidogenic protein levels is missing. However, it is possible that longer term exposure could result in reductions in the protein levels. For example, in cultured adrenocortical cells, long term (48 hours, 1 μM) exposure to etomidate was able to reduce the steroidogenic output to less than 10%, well over the simple competitively inhibition of enzyme themselves, suggesting a combination effects of reduction in protein levels and of direct inhibition in enzyme activity of CYP11A1 [25]

Etomidate is widely used in clinics. To maintain the ideal anesthesia, a blood level of 300~500 ng/ml (1.22–2.04 μM) is required. When its blood level reaches 150~300 ng/ml (0.61–1.22 μM), etomidate causes sedentary effects. In this concentration range, etomidate apparently is also capable of interfering androgen formations by inhibiting the enzyme activities. Whether such inhibitions might also happen to human boys who contain comparable developmental stages of immature Leydig cells as we used in this study deserves further study.

## Supporting Information

S1 Fig Representative thin layer chromatograph and radiometer scanning graph of HSD17B3. Rat testis HSD17B3 activity was measured by adding androstenedione (D4) and the formation of product of testosterone (T) by rat testis microsome.
(EPS)

S2 Fig The inhibitory mode of etomidate on HSD3B1 for NAD^+^. Rat testis HSD3B1 activity was measured with various concentration of etomidate (ETO). Lineweaver–Burk plot for HSD3B1 in the presence of NAD+. Mean ± SEM, n = 4.
(EPS)

## Abbreviations

25R-OHC: 25R-OH-cholesterol
D4: Androstenedione
DIOL: 5α-androstane-3α,17β-diol
DHT: Dihydrotestosterone
T: Testosterone
P4: Progesterone
P5: Pregnenolone
17OHP4: 17-hydroxy-Progesterone
CYP11A1: P450 cholesterol side chain cleavage enzyme
HSD3B: 3β-hydroxysteroid dehydrogenase
CYP17A1: 17α-hydroxylase/C17,20-lyase
HSD17B3: 17β-hydroxysteroid dehydrogenase
SRD5A1, encoded by Srd5a1: 5α-reductase 1
HSD3A, encoded by Akr1c14: 3α-hydroxysteroid dehydrogenase
LH: Luteinizing hormone
LHCGR, encoded by *Lhcgr*: LH receptor
8Br-cAMP: 8-Bromoadenosine 3',5'-cyclic monophosphate
SCARB1, encoded by *Scarb1*: Scavenger receptor class B member 1
StAR, encoded by *Star*: Steroidogenic acute regulatory protein
PBS: Phosphate buffered saline

## References

[1] Doenicke A. Etomidate, a new intravenous hypnotic. Acta anaesthesiologica Belgica. 1974;25(3):307–15. . 4143092

[2] Tomlin SL, Jenkins A, Lieb WR, Franks NP. Stereoselective effects of etomidate optical isomers on gamma-aminobutyric acid type A receptors and animals. Anesthesiology. 1998;88(3):708–17. Epub 1998/04/02. . 9523815 10.1097/00000542-199803000-00022

[3] Husain SS, Ziebell MR, Ruesch D, Hong F, Arevalo E, Kosterlitz JA, et al 2-(3-Methyl-3H-diaziren-3-yl)ethyl 1-(1-phenylethyl)-1H-imidazole-5-carboxylate: a derivative of the stereoselective general anesthetic etomidate for photolabeling ligand-gated ion channels. J Med Chem. 2003;46(7):1257–65. Epub 2003/03/21. 10.1021/jm020465v . 12646036

[4] Gooding JM, Corssen G. Etomidate: an ultrashort-acting nonbarbiturate agent for anesthesia induction. Anesth Analg. 1976;55(2):286–9. Epub 1976/03/01. . 943993 10.1213/00000539-197603000-00035

[5] Forman SA. Clinical and molecular pharmacology of etomidate. Anesthesiology. 114(3):695–707. Epub 2011/01/26. 10.1097/ALN.0b013e3181ff72b5 21263301 PMC3108152

[6] Ledingham IM, Watt I. Influence of sedation on mortality in critically ill multiple trauma patients. Lancet 1983;321 S0140-6736(83)92712-5. 10.1016/s0140-6736(83)92712-5 6134053

[7] Wagner RL, White PF, Kan PB, Rosenthal MH, Feldman D. Inhibition of adrenal steroidogenesis by the anesthetic etomidate. N Engl J Med. 1984;310(22):1415–21. Epub 1984/05/31. 10.1056/NEJM198405313102202 . 6325910

[8] Wagner RL, White PF, Kan PB, Rosenthal MH, Feldman D. Inhibition of adrenal steroidogenesis by the anesthetic etomidate. New England Journal of Medicine. 1984 310:1415–21. 6325910 10.1056/NEJM198405313102202

[9] Schulte HM, Benker G, Reinwein D, Sippell WG, Allolio B. Infusion of low dose etomidate: correction of hypercortisolemia in patients with Cushing's syndrome and dose-response relationship in normal subjects. J Clin Endocrinol Metab. 1990;70(5):1426–30. Epub 1990/05/01. 10.1210/jcem-70-5-1426 . 2159485

[10] Diago MC, Amado JA, Otero M, Lopez-Cordovilla JJ. Anti-adrenal action of a subanaesthetic dose of etomidate. Anaesthesia. 1988;43(8):644–5. Epub 1988/08/01. . 3421456 10.1111/j.1365-2044.1988.tb04148.x

[11] Wagner RL, White PF. Etomidate inhibits adrenocortical function in surgical patients. Anesthesiology. 1984;61(6):647–51. Epub 1984/12/01. . 6095700 10.1097/00000542-198412000-00003

[12] Zolle IM, Berger ML, Hammerschmidt F, Hahner S, Schirbel A, Peric-Simov B. New selective inhibitors of steroid 11beta-hydroxylation in the adrenal cortex. Synthesis and structure-activity relationship of potent etomidate analogues. J Med Chem. 2008;51(7):2244–53. Epub 2008/03/20. 10.1021/jm800012w . 18348518

[13] Heytens L, Devroey P, Camu F, Van Steirteghem AC. Effects of etomidate on ovarian steroidogenesis. Hum Reprod. 1987;2(2):85–90. Epub 1987/02/01. . 3108307 10.1093/oxfordjournals.humrep.a136506

[14] Lambert A, Mitchell R, Robertson WR. Biopotency and site of action of drugs affecting testicular steroidogenesis. J Endocrinol. 1987;113(3):457–61. 2957454 10.1677/joe.0.1130457

[15] Ye L, Su ZJ, Ge RS. Inhibitors of testosterone biosynthetic and metabolic activation enzymes. Molecules. 2011;16(12):9983–10001. Epub 2011/12/06. molecules16129983 [pii] 10.3390/molecules16129983 . 22138857 PMC6264586

[16] Payne AH, Abbaszade IG, Clarke TR, Bain PA, Park CH. The multiple murine 3beta-hydroxysteroid dehydrogenase isoforms: structure, function, and tissue and developmentally specific expression. Steroids. 1997;62(1):169–75. 9029733 10.1016/s0039-128x(96)00177-8

[17] Ge RS, Hardy MP. Variation in the end products of androgen biosynthesis and metabolism during postnatal differentiation of rat Leydig cells. Endocrinology. 1998;139(9):3787–95. Epub 1998/09/02. . 9724031 10.1210/endo.139.9.6183

[18] Payne AH, Wong KL, Vega MM. Differential effects of single and repeated administrations of gonadotropins on luteinizing hormone receptors and testosterone synthesis in two populations of Leydig cells. J Biol Chem. 1980;255(15):7118–22. Epub 1980/08/10. . 6248547

[19] Ge RS, Gao HB, Nacharaju VL, Gunsalus GL, Hardy MP. Identification of a kinetically distinct activity of 11beta-hydroxysteroid dehydrogenase in rat Leydig cells. Endocrinology. 1997;138(6):2435–42. Epub 1997/06/01. . 9165033 10.1210/endo.138.6.5165

[20] Hu GX, Zhou HY, Li XW, Chen BB, Xiao YC, Lian QQ, et al The (+)- and (-)-gossypols potently inhibit both 3beta-hydroxysteroid dehydrogenase and 17beta-hydroxysteroid dehydrogenase 3 in human and rat testes. J Steroid Biochem Mol Biol. 2009;115(1–2):14–9. Epub 2009/05/12. S0960-0760(09)00056-9 [pii] 10.1016/j.jsbmb.2009.02.004 . 19429456

[21] Lin H, Ge RS, Chen GR, Hu GX, Dong L, Lian QQ, et al Involvement of testicular growth factors in fetal Leydig cell aggregation after exposure to phthalate in utero. Proc Natl Acad Sci U S A. 2008;105(20):7218–22. Epub 2008/05/13. 0709260105 [pii] 10.1073/pnas.0709260105 18469139 PMC2438230

[22] Guo J, Zhou H, Su Z, Chen B, Wang G, Wang CQ, et al Comparison of cell types in the rat Leydig cell lineage after ethane dimethanesulfonate treatment. Reproduction. 2013;145(4):371–80. Epub 2013/04/17. 145/4/371 [pii] 10.1530/REP-12-0465 . 23587774

[23] Hanukoglu I, Gutfinger T, Haniu M, Shively JE. Isolation of a cDNA for adrenodoxin reductase (ferredoxin-NADP+ reductase). Implications for mitochondrial cytochrome P-450 systems. Eur J Biochem. 1987;169(3):449–55. Epub 1987/12/15. . 3691502 10.1111/j.1432-1033.1987.tb13632.x

[24] Hanukoglu I, Jefcoate CR. Mitochondrial cytochrome P-450scc. Mechanism of electron transport by adrenodoxin. J Biol Chem. 1980;255(7):3057–61. Epub 1980/04/10. . 6766943 10.1016/S0021-9258(19)85851-9

[25] Rijk JC, Peijnenburg AA, Blokland MH, Lommen A, Hoogenboom RL, Bovee TF. Screening for modulatory effects on steroidogenesis using the human H295R adrenocortical cell line: a metabolomics approach. Chem Res Toxicol. 2012;25(8):1720–31. 10.1021/tx3001779 22768806

